# Lesions of lateral or central amygdala abolish aversive Pavlovian-to-instrumental transfer in rats

**DOI:** 10.3389/fnbeh.2014.00161

**Published:** 2014-05-07

**Authors:** Vincent D. Campese, Jeanny Kim, Gabriel Lázaro-Muñoz, Lashawn Pena, Joseph E. LeDoux, Christopher K. Cain

**Affiliations:** ^1^Center for Neural Science, New York UniversityNew York, NY, USA; ^2^Emotional Brain Institute, Nathan Kline Institute for Psychiatric ResearchOrangeburg, NY, USA; ^3^Department of Psychology, Hunter College, CUNYNew York, NY, USA; ^4^Child and Adolescent Psychiatry, New York University School of MedicineNew York, NY, USA

**Keywords:** Pavlovian, instrumental, transfer, avoidance, shuttling, rat, amygdala

## Abstract

Aversive Pavlovian conditioned stimuli (CSs) elicit defensive reactions (e.g., freezing) and motivate instrumental actions like active avoidance (AA). Pavlovian reactions require connections between the lateral (LA) and central (CeA) nuclei of the amygdala, whereas AA depends on LA and basal amygdala (BA). Thus, the neural circuits mediating conditioned reactions and motivation appear to diverge in the amygdala. However, AA is not ideal for studying conditioned motivation, because Pavlovian and instrumental learning are intermixed. Pavlovian-to-instrumental transfer (PIT) allows for the study of conditioned motivation in isolation. PIT refers to the ability of a Pavlovian CS to modulate a separately-trained instrumental action. The role of the amygdala in aversive PIT is unknown. We designed an aversive PIT procedure in rats and tested the effects of LA, BA, and CeA lesions. Rats received Pavlovian tone-shock pairings followed by Sidman shock-avoidance training. PIT was assessed by comparing shuttling rates in the presence and absence of the tone. Tone presentations facilitated instrumental responding. Aversive PIT was abolished by lesions of LA or CeA, but was unaffected by lesions of BA. These results suggest that LA and CeA are essential for aversive conditioned motivation. More specifically, the results are consistent with a model of amygdala processing in which the CS is encoded in the LA and then, via connections to CeA, the motivation to perform the aversive task is enhanced. These findings have implications for understanding the contribution of amygdala circuits to aversive instrumental motivation, but also for the relation of aversive and appetitive behavioral control.

## Introduction

In studies of Pavlovian threat conditioning (PTC) a conditioned stimulus (CS; e.g., tone) is paired with an aversive unconditioned stimulus (US; e.g., footshock). Conditioning transforms the CS into a threat which elicits innate defensive reactions [e.g., freezing; see (LeDoux, 2000)]. PTC depends on connections between the lateral (LA) and central (CeA) nuclei of the amygdala (Jimenez and Maren, 2009). Pavlovian CSs can also motivate instrumental actions. In active avoidance (AA), the CS signals when a response will prevent shock (Cain and LeDoux, 2008). Although the amygdala is also important for AA (Sarter and Markowitsch, 1985), the contribution of amygdala nuclei is quite different from PTC. Early in training, lesions of LA or basal amygdala (BA) disrupt AA, but CeA lesions do not (Choi et al., 2010). And unlike PTC, AA becomes amygdala-independent with overtraining (Poremba and Gabriel, 1999; Lazaro-Munoz et al., 2010). Similar findings have been obtained with escape from fear (or EFF), a related task (Amorapanth et al., 2000). These findings support a model where (1) LA is critical for CS-US learning, (2) intraamygdala connections between LA and CeA mediate Pavlovian reactions, and (3) LA and BA connections mediate non-habitual instrumental responding (Amorapanth et al., 2000; Cain et al., 2013).

However, AA is not ideal for studying the neural mechanisms of conditioned motivation because the Pavlovian CS contributes to both reinforcement and motivation of the instrumental response (Cain and LeDoux, 2008). While EFF tasks begin to separate these components, Pavlovian-to-instrumental transfer (PIT) tasks are an even more effective way to study motivation processes in isolation (Estes, 1948; Lovibond, 1983). In PIT tasks, Pavlovian and instrumental conditioning occur separately. During the transfer test, CS-elicited changes in response rate serve as an index of conditioned motivation. While the neural basis of appetitive PIT has been studied extensively (see Hall et al., 2001; Holland and Gallagher, 2003; Corbit and Balleine, 2005; Balleine and Killcross, 2006), virtually nothing is known about the brain mechanisms of aversive PIT. Some have drawn conclusions about the neural control of Pavlovian and instrumental interactions in aversive conditioning based on findings from studies evaluating conditioned suppression. However, these studies are insufficient in providing information on aversive PIT because the behavioral phenomena are quite different. While the CS invigorates instrumental responding in classic PIT tasks, it attenuates responding in suppression tasks. We therefore designed an aversive PIT task analogous to the classic version of the phenomenon (see Campese et al., 2013) and have used it to evaluate the importance of amygdala nuclei in PIT. In our assay, rats received PTC followed by unsignaled, two-way, Sidman active avoidance (USAA). During PIT testing, rats responded in extinction, and USAA response rates were recorded during pre-CS and CS intervals. Similar to appetitive PIT, CS presentations enhanced instrumental responding. Electrolytic lesions of LA, BA, or CeA were then placed and subsequently evaluated for the resulting changes in PIT. Since both AA and EFF depend on aversive conditioned motivation and require LA and BA, but not CeA (Amorapanth et al., 2000; Choi et al., 2010; Lazaro-Munoz et al., 2010), we hypothesized that aversive PIT would also depend on LA and BA, but not CeA.

## Materials and methods

### Subjects

Male Sprague-Dawley rats (Hilltop Lab Animals Inc., Scottsdale, PA) weighing approximately 300 g at the start of the study were used as subjects. Experiments were conducted at two locations, the Nathan Kline Institute for Psychiatric Research (NKI; Orangeburg, NY) and New York University (NYU; New York, NY). Rats at NKI were housed 2/cage whereas rats at NYU were singly housed. Otherwise, housing conditions were identical. Rats had free access to food and water and were maintained on a 12:12 light:dark schedule. All procedures were performed in accordance with National Institutes of Health guidelines and were approved by NKI and NYU Animal Care and Use Committees.

### Apparatus

Threat conditioning occurred in standard conditioning boxes (H10-11R-TC: Coulbourn Instruments, Whitehall PA). USAA training and PIT testing occurred in two-way shuttleboxes (H10-11R-SC: Coulbourn Instruments). Conditioning boxes and shuttleboxes were housed in sound-attenuating chambers (model H10-24A). All boxes were equipped with house lights, infrared indicator lights, video cameras and 8 Ohm speakers to deliver the tone CS (generated by a programmable tone generator: model A12-33). The scrambled footshock US was delivered through stainless steel grid floors (model H10-11R-TC-SF). Shuttleboxes were also equipped with infrared beams to automatically detect movement between the two chamber sides.

### Procedure

**Figure 1:**
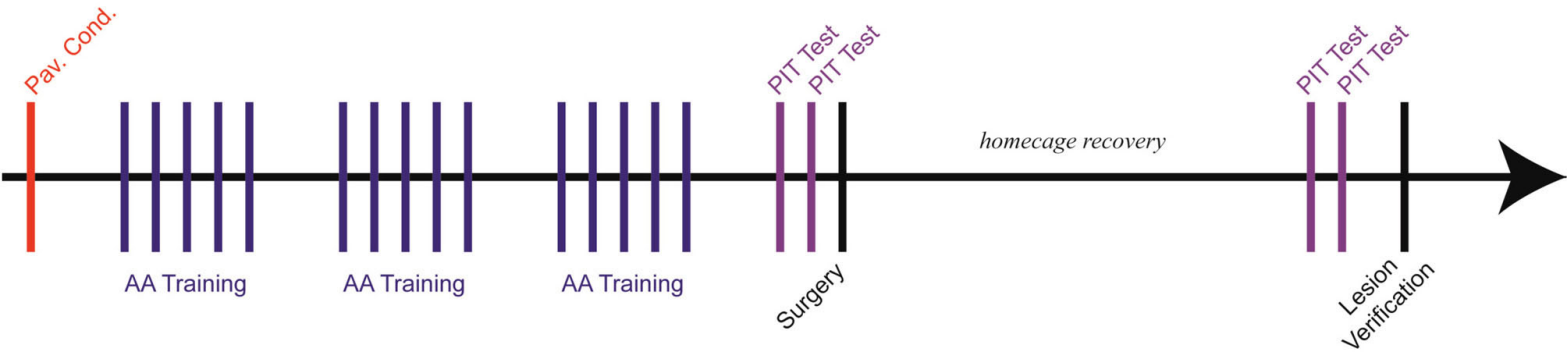
**Experimental timeline (~ 45 days beginning to end)**. Each vertical line represents a single session. Subjects were given only one session per day with approximately 24 h between sessions. Five USAA sessions were run per week, typically, one per weekday with weekends off.

Six phases comprised the experiment, and all procedures have been described in detail elsewhere (Amorapanth et al., 2000; Lazaro-Munoz et al., 2010; Campese et al., 2013): (1) Pavlovian threat conditioning (PTC), (2) unsignaled Sidman active avoidance conditioning (USAA), (3) pre-lesion PIT tests, (4) lesion surgery, (5) post-lesion PIT tests and (6) lesion verification. Subjects received one behavioral session per day, excluding weekends and the 2-week surgical recovery period (see Figure 1 for experimental timeline).

#### Pavlovian threat conditioning (PTC)

Rats received three pairings of the CS (30 s, 5 kHz, 80 dB tone) and US (0.7 mA × 1 s footshock) with 3-min intervals preceding and following the pairings.

#### Unsignaled sidman active avoidance (USAA) training

Within 3 days following the PTC session, rats began 15 days (5 days per week) of USAA training where every shuttling response (movement to the opposite chamber side) delayed the delivery of the US (0.5 s footshock) by 30 s (R–S or response-shock interval). In the absence of shuttling, the US was delivered every 5 s (S–S or shock-shock interval). Avoidance responses were defined as shuttles during the R–S interval; shuttles during the S–S interval were considered escape responses. All shuttles were marked by a brief feedback stimulus (house lights blink off for 0.3 s). After session 10 of USAA training, poor avoiders (rats failing to exhibit 20 or more avoidance responses for two consecutive sessions, see Lazaro-Munoz et al. (2010)) were excluded from further training and PIT testing. Only good avoiders continued USAA training for another five sessions. Due to a miscommunication, the US intensity for USAA training was 1mA for studies conducted at NKI and 0.7 mA for studies conducted at NYU.

#### Pavlovian-to-instrumental transfer (PIT) testing

Following USAA training, subjects received two daily PIT tests, then surgery, and then another two PIT tests following recovery. All PIT test sessions were identical and involved a single presentation of the aversive CS in the shuttleboxes while rats shuttled under extinction (US presentations absent, response feedback present). For each individual, the CS presentation was triggered when the shuttling rate fell below two responses per minute (RPMs) for two full minutes. Previous work found that PIT effects were greatest when baseline response rates were low (~2 RPMs), but not absent (Campese et al., 2013). Since rats vary greatly in their rates of USAA extinction, this protocol ensured similar baseline response rates when PIT was assessed. Additionally, since some rats freeze when initially placed in the shuttleboxes, the CS trigger was disabled for the first 15 min of USAA extinction. Importantly, because the high-freezing poor-avoiding rats were eliminated from further experimentation following session 10 of USAA, freezing during the test phase was minimized. While freezing to the USAA context is already extremely low in good avoiders given the amount of training subjects underwent before tests (see Lazaro-Munoz et al., 2010) this mandatory baseline further ensured that freezing responses did not influence PIT. Once triggered, the CS presentation remained on until 10 shuttles were performed. Immediately after the 10th shuttle response, the CS was terminated, the houselight turned off and the session ended. For each rat in each test, a PIT score was calculated by the following equation: (shuttling rate during the CS/shuttling rate during an equivalent Pre-CS period)^*^100. Rats were matched into lesion treatment groups based on their performance in the first two pre-lesion PIT tests. Matching is an accepted technique used to create equivalent groups, with comparable central tendency, and variability. Any individual differences in PIT would be expected to normally distribute across the groups using this technique. Initial USAA rates (shuttling during the 1st min of PIT testing) and Peak USAA rates (maximal pre-CS rate during PIT testing) were also recorded to assess the USAA memory during repeated PIT testing.

### Surgery

Rats were anesthetized with ketamine and xylazine (i.p., 100 mg/kg; 6.0 mg/kg, Phoenix Pharmaceutical), and placed in a stereotaxic apparatus (David Kopf Instruments, Tujunga, CA). Small burr holes were drilled above the targeted brain area. A stainless steel monopolar electrode covered with epoxy (exposed tip of 500 μm for CeA and 250 μm for LA and B lesions; model NE-300X and SNEX-300X, David Kopf Instruments) was lowered through an incision in the dura into the target site. Lesions were created with a lesion maker (model 53500, Ugo Basile, Italy) by passing current (+0.5 mA) of different durations as previously described (see (Lazaro-Munoz et al., 2010) Table 1 for coordinates and current parameters). Sham animals underwent the same procedure, but no current was passed through the electrode. Animals recovered in their homecages for 14 days following surgery.

### Lesion verification

At the completion of behavioral testing, rats were given an anesthetic overdose and perfused transcardially with 10% phosphate buffered formalin. Brains were removed and stored in 10% phosphate-buffered formalin and 30% sucrose for at least 3 days and were then cut in 50 μm sections using a freezing microtome (every other section was collected). Nissl stains were then performed and tissue images were collected (Nikon Microphot-FXA). Damage to target brain regions and adjacent areas was assessed using a rat brain atlas as a guide (Paxinos and Watson, 2005).

### Statistical analysis

Data in Figure 3 are descriptive examples and were not subjected to statistical analysis. Otherwise, data represent group means (± s.e.m.) and were analyzed with two-way Phase (pre-lesion, post-lesion) × Group (Sham, Amygdala, LA, BA, CeA) ANOVAs treating phase as a repeated measure (GraphPad Prism v5.01, GraphPad Software Inc., La Jolla, CA). Planned post-hoc comparisons of group means vs. sham-operated controls were analyzed using Bonferroni's Multiple Comparison test. Differences were considered significant if p values were less than 0.05.

## Results

**Figure 2:**
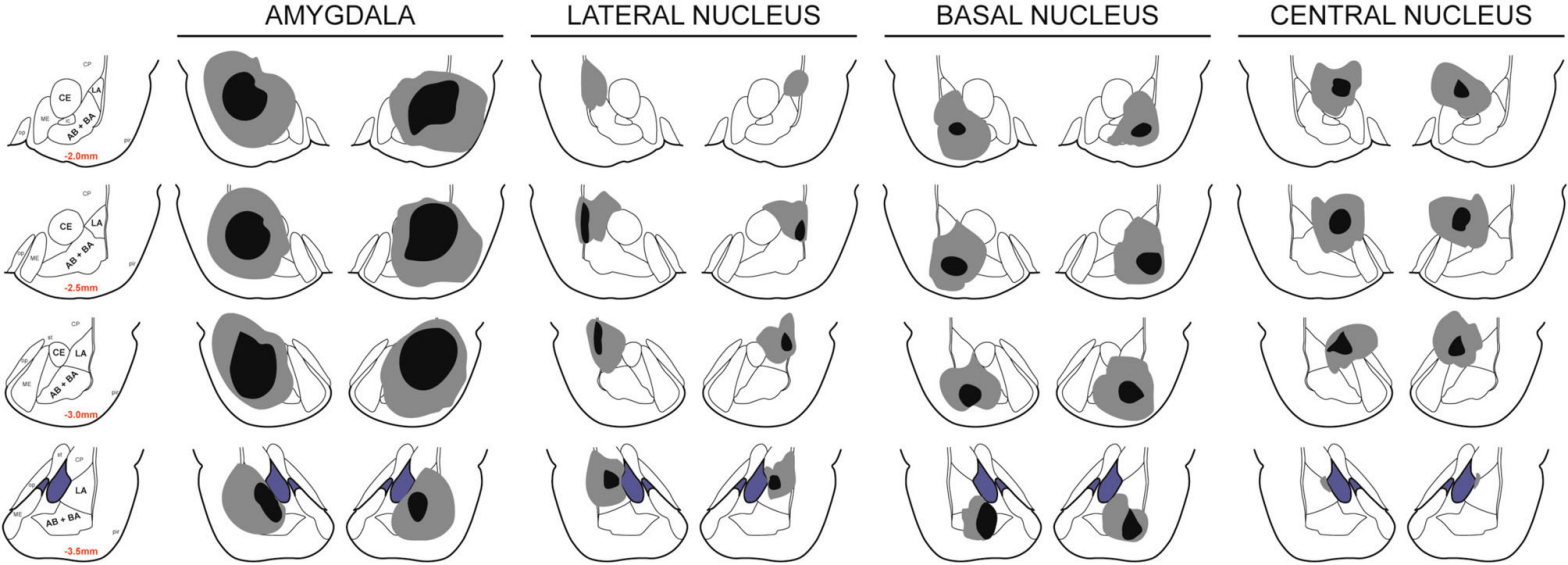
**Lesion placements**. Shaded areas represent the greatest (gray) and least (black) extent of electrolytic lesions. Red numbers reflect distance from bregma in millimeters. Brain slides adapted from Paxinos and Watson (2005) with permission from Elsevier.

This study included 100 rats, 27 of which were part of the sham groups. Figure 2 depicts the extent of acceptable lesions in the final dataset. LA, BA and CeA were targeted in 16, 47, and 11 rats respectively. Forty-three rats were excluded because of insufficient bilateral damage to the target nucleus or excessive damage to adjacent, non-targeted amygdala regions. Eight rats from one batch of BA lesions had significant bilateral damage to LA, BA, and CeA. It is not clear what caused these excessively large lesions, perhaps an insufficiently insulated electrode. These rats were re-categorized as “amygdala” lesions and data are presented for comparison with the other groups. Five additional rats were excluded from analyses because of a failure to shuttle during the pre-CS period during post-surgery PIT tests. This behavioral profile did not seem to be related to lesions as two rats were from the sham group, two were from the BA lesion group and one was from the CeA lesion group. Thus, the final groups included 25 shams, eight amygdala lesions, eight LA lesions, five BA lesions and six CeA lesions.

**Figure 3:**
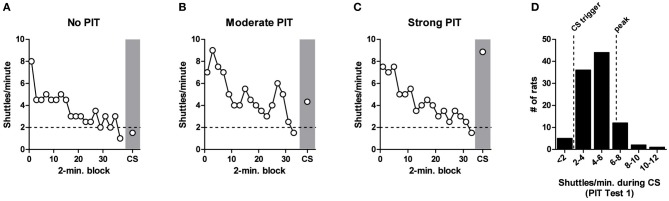
**Range of behavior during aversive PIT testing. (A–C)**, White circles show shuttling rate during AA extinction in 2-min blocks for three individual rats. When shuttling rate dips below 2 responses/minute (dashed horizontal line) for 2 min, the CS is triggered (gray shading). Examples of No PIT, Moderate PIT and Strong PIT are shown. **(D)**, Histogram showing frequency distribution of PIT effects for 100 rats during PIT Test 1. Vertical dashed lines mark the CS trigger (2 RPMs) and the peak shuttling rate during extinction for this batch of rats (mean = 6.2 RPMs).

Figure 3 shows three examples of shuttling rates to illustrate the range of behavior during PIT testing with our protocol (selected from the pre-lesion PIT tests). In each case, shuttling rates were initially high and then decreased as animals responded in extinction. Once shuttling rates dipped below two RPMs for 2 min, the CS was presented. In rare cases the CS had no effect on shuttling rate (e.g., Figure 3A), or, on the opposite extreme, the CS boosted responding beyond peak rates (e.g., Figure 3C). However, in the vast majority of cases (74%), the threat CS reinvigorated shuttling to a level between the pre-CS baseline and the peak rate observed during USAA extinction (e.g., Figure 3B). This notion is supported by a frequency distribution for shuttling rates during the CS (Figure 3D).

PIT effects and USAA rates were analyzed using Group (Sham, Amygdala, LA, BA, CeA) × Phase (pre-lesion, post-lesion) ANOVAs with planned post-hoc contrasts between individual lesion groups and shams. Since there were no significant differences between PIT tests 1 and 2 during either phase, data for individuals were averaged to obtain a single pre-lesion and post-lesion score for each dependent measure. There were also no differences between Sham groups targeting LA, BA, or CeA, and these animals were combined into a single group for the final analysis. Lastly, although different US intensities were inadvertently used at NKI and NYU for USAA training (1.0 and 0.7 mA, respectively), we found no PIT differences between the institutions and animals were combined into single Sham, Amygdala, LA, BA, and CeA groups for the final analysis.

**Figure 4:**
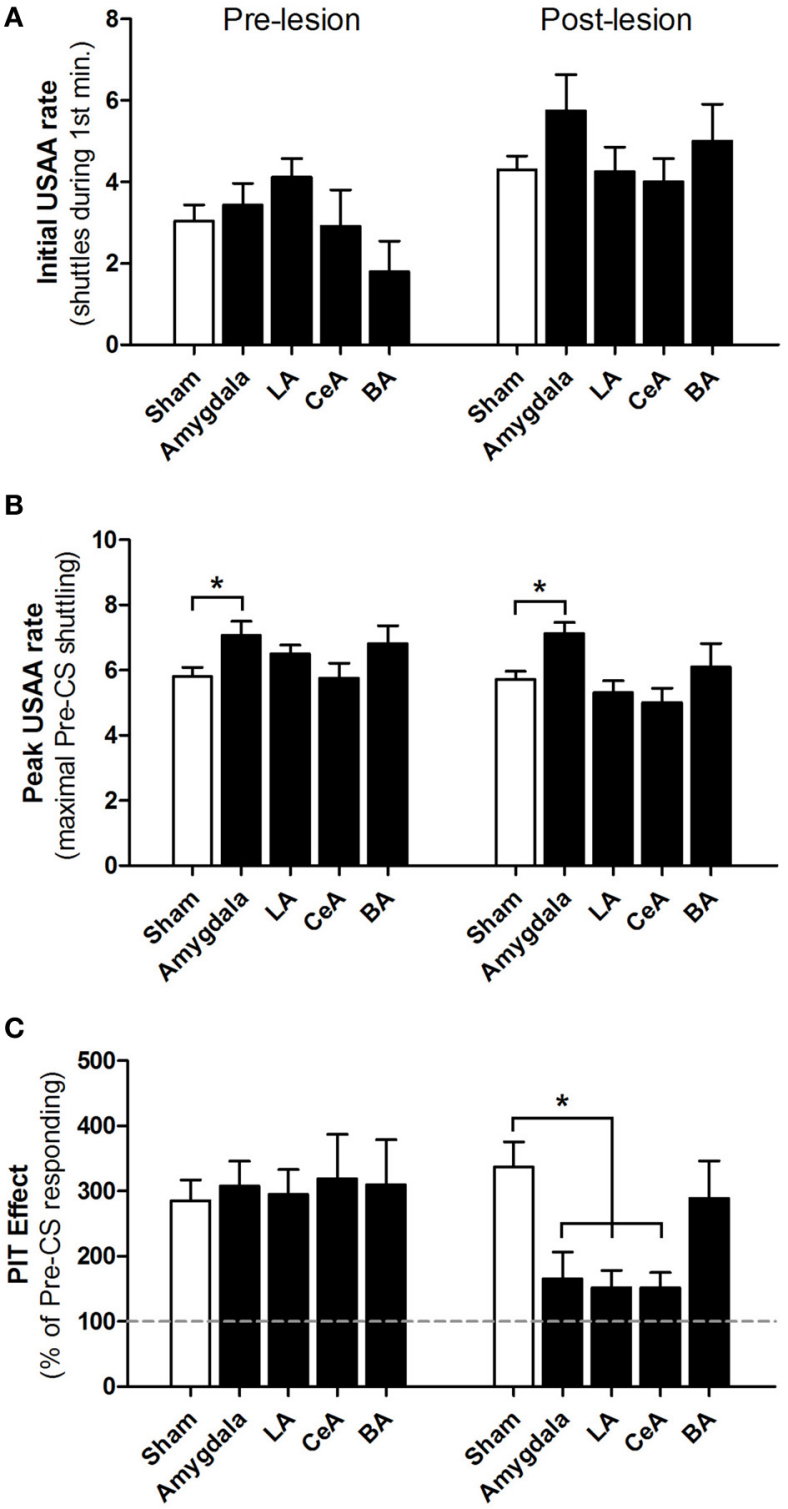
**Effect of amygdala lesions on USAA responding and aversive PIT**. Each graph shows pre-lesion (mean of PIT tests 1 and 2) and post-lesion (mean of PIT tests 3 and 4) testing phases. **(A)** Number of shuttles during the first minute of PIT testing for Shams (*n* = 25), Amygdala lesions (*n* = 8), LA lesions (*n* = 8), BA lesions (*n* = 5) and CeA lesions (*n* = 6). **(B)** Peak shuttling rate during PIT testing, defined as the greatest number of shuttles for a single minute during the pre-CS USAA extinction phase. **(C)** Aversive PIT effect: shuttling rate during CS presentations, expressed as a percentage of pre-CS responding. The gray dashed line at 100% represents an absence of PIT (no facilitation of responding). ^*^p < 0.05 vs. Sham-operated controls.

Figure 4 shows USAA and PIT measures during the pre-lesion and post-lesion phases for all groups. USAA rates at the start of PIT testing did not differ between the groups in either phase (Figure 4A); both the Group [*F*_(4, 47)_ = 0.9, *p* = 0.46] and Group × Phase interaction [*F*_(4, 47)_ = 2.0, *p* = 0.12] were insignificant. Rats in all groups showed higher initial USAA rates during post-lesion tests, perhaps due to extinction of Pavlovian freezing to contextual cues with repeated PIT testing; the effect of Phase was significant [*F*_(1, 47)_ = 20.5, *p* < 0.01]. Peak USAA rates, before the CS was presented, were comparable for most groups during both the pre- and post-lesion phases (Figure 4B). However, the effects of Group [*F*_(4, 47)_ = 4.0, *p* < 0.01] and Phase [*F*_(1, 47)_ = 4.6, *p* = 0.04] were statistically significant. Post-hoc contrasts revealed that peak USAA rates differed from Shams in the Amygdala-lesioned group only (*p* < 0.05 for both pre- and post-lesion tests). The Group × Phase interaction was not significant [*F*_(4, 47)_ = 1.1, *p* = 0.36]. Thus, this difference is likely due to imperfect matching rather than an effect of lesions. For the critical PIT measure, there were no differences between groups during the pre-lesion phase, but large differences after lesions (Figure 4C); the Group × Phase interaction was highly significant [*F*_(4, 47)_ = 4.9, *p* < 0.01]. *Post-hoc* contrasts revealed no PIT differences between groups in the pre-lesion phase (*p* values > 0.05). However, in the post-lesion phase, Amygdala-, LA- and CeA-lesioned groups all showed significantly weaker PIT than Shams (*p* values <0.05). In contrast, PIT was intact in animals with BA lesions (*p* >0.05 vs. Shams). Post-lesion contrasts between the BA-lesioned group and the Amygdala-, LA- and CeA-lesioned groups failed to reach statistical significance (*p* values >0.05). These results were confirmed with additional tests. Direct comparisons (parametric and non-parametric) between pre- and post-surgical PIT scores agree with the *post hoc* analyses. Together, these results indicate that Amygdala, LA and CeA lesions impair aversive PIT without affecting baseline USAA performance.

## Discussion

The present experiments used a novel behavioral protocol (Campese et al., 2013) to evaluate the effects of LA, BA or CeA lesions on aversive PIT in rats. The development of this task was informed by earlier studies of CS facilitated avoidance behavior (e.g., Overmier and Payne, 1971). In the first experimental phase, Pavlovian conditioning established the tone CS as a threat. In the second phase, USAA training was used to generate a steady rate of instrumental responding. During the critical PIT testing phases, animals were allowed to respond in USAA-extinction and were then presented with the Pavlovian CS, normally resulting in enhanced instrumental responding. This basic procedure and behavioral effect mirrors that seen with appetitive PIT studies, where food-paired cues increase food-rewarded instrumental response rates (e.g., Crombag et al., 2008). Note that associative Pavlovian conditioning is required to achieve the aversive PIT effect; both naïve and unpaired controls fail to show facilitation of USAA responding during the PIT test phase (Campese et al., 2013).

In contrast to our hypothesis that LA and BA, but not CeA, would be required, we found that electrolytic lesions of LA or CeA, but not BA, impaired aversive PIT. Importantly, none of the lesions impaired the baselines response, USAA responding, after 15 training sessions. This result agrees with our previous work (Lazaro-Munoz et al., 2010). suggesting that USAA is amygdala independent by day 15 of training, which is crucial for the purpose of this study. It is worth emphasizing that the LA, BA, and CeA are not needed for normal USAA behavior at the time PIT testing occurred. This means that the shuttling response itself is not dependent upon these structures given extended USAA training. This assertion is supported by our data demonstrating that our lesioned subjects show no initial or peak USAA responding impairments (Figures 4A,B). Given this knowledge (see also Lazaro-Munoz et al., 2010) together with our use of matching surgical groups based on PIT scores, it is, therefore, highly unlikely that unequal distributions of subjects that depend on one or more these structures for shuttling are present in our surgical conditions. This pattern of lesion results suggests that LA and CeA are both required, and that connections between these allow the CS to facilitate responding. At present, it is unclear how activity in this pathway can mediate both Pavlovian reactions, like freezing, and facilitation of instrumental actions, like shuttling. However, different cell populations in CeA are known to mediate different conditioned responses (Viviani et al., 2011), including active and passive defensive reactions (Gozzi et al., 2010). Perhaps PIT is favored over freezing when an AA response is available, via some prefrontal or hippocampal gating mechanism in CeA (Ji and Maren, 2007; Sotres-Bayon and Quirk, 2010).

The dependence of PIT on CeA and not BA was surprising given that BA, but not CeA, is involved in other instrumental tasks that we have studied, including AA and EFF (Amorapanth et al., 2000; Choi et al., 2010). An important procedural similarity between AA and EFF that is not shared by PIT is that the CS is present while the instrumental response is being acquired and in fact its termination serves as a conditioned negative reinforcer that supports the learning. For AA and EFF each trial is initiated by the onset of the tone CS, while in PIT the tone CS is not presented during the USAA instrumental phase—the PIT phenomenon is entirely expression based. The difference between lesion effects in PIT and AA/EFF suggests that an aversive CS can motivate instrumental actions in different ways, via different neural pathways—perhaps these pathways are recruited depending on how the instrumental response was acquired (i.e., in the presence or absence of a discreet Pavlovian CS). The contribution of the tone CS to instrumental acquisition in AA and EFF but not to USAA may provide some explanation for the differences in lesion results among the studies using these tasks.

A major goal of this project was to compare aversive and appetitive PIT findings in studies involving brain manipulations. While our PIT task is analogous to simple appetitive PIT, the studies examining the neural circuits in this field have evolved into something more complex. Corbit et al. (2001), Corbit and Balleine (2005) have found that PIT can be driven by shared motivational and sensory components for food USs associated with both Pavlovian CSs and instrumental responses. More simply put, a CS paired with food of some variety will enhance overall responding (i.e., non-specifically) in a two-lever choice test. Each lever in this test had also been previously reinforced with food, but, importantly, not the same food as the CS signaled. This is an example of general PIT, distinct from sensory specific PIT in which a CS enhances responding only on the lever that was reinforced with the same food US as the CS signals. General PIT has been shown to involve the CeA while specific PIT requires the BLA (or basolateral amygdala—these studies did not treat BA and LA as distinct structures). Balleine and Killcross (2006) have argued that the PIT findings go against the traditional view of amygdala processing known as the serial model (LeDoux, 2000). They propose a parallel model in which the CeA processes motivational value while the BLA is involved in sensory encoding. While quite simple by comparison in terms of experimental design, our aversive task is likely an instance of general PIT, which according to this model should be impaired only by CeA lesions (see Hall et al., 2001; Holland and Gallagher, 2003; Campese et al., 2013). Because the LA and the CeA are both required, this suggests that these structures are not working in parallel, but rather serially in our aversive PIT task. While evidence suggests that appetitive and aversive processing in the amygdala are not accomplished in the same way (Gallagher and Holland, 1994; Baxter and Murray, 2002) another potential explanation for the difference in PIT lesion results is that LA may have been insufficiently damaged in appetitive studies.

Due to the treatment of BA and LA as a single structure (i.e., the BLA) in appetitive conditioning studies, subjects meeting lesion criteria for this group could potentially have an intact dorsal LA. Aversive studies have shown that the dorsal part of LA is most important for Pavlovian conditioning (Repa et al., 2001; Rosenkranz and Grace, 2002; Han et al., 2007, 2009). Thus, it is possible that appetitive general PIT also depends on serial connections between LA and CeA, but that LA was insufficiently damaged in past studies targeting BLA. However, we view this explanation as less likely since one study of appetitive general PIT clearly damaged dorsal LA even with the smallest lesions (Holland and Gallagher, 2003).

In conclusion, we have developed a novel aversive PIT protocol in rats where threatening CSs facilitate the expression of USAA responding. Lesions of LA or CeA, but not BA, impair aversive PIT. These results are consistent with a serial model of amygdala CS processing, although they do not contradict the notion that parallel processing occurs with appetitive conditioning, or with overtraining of the Pavlovian CS-US association (Killcross et al., 1997). Considered with findings from PTC, AA and EFF studies, these results also suggest that aversive conditioned motivation depends on different outputs of LA, depending on whether the Pavlovian CS is part of the instrumental associative structure. Ultimately, more complex protocols will be necessary to evaluate outcome-specific vs. general aversive PIT in the same animals, and to clarify whether the amygdala processes aversive and appetitive CSs differently. Interestingly, a recent study demonstrated both general and outcome-specific aversive PIT in humans (Nadler et al., 2011). Thus, aversive PIT studies are relevant to human behavior and future human imaging studies may help resolve the debate regarding serial vs. parallel processing of Pavlovian information.

### Conflict of interest statement

The authors declare that the research was conducted in the absence of any commercial or financial relationships that could be construed as a potential conflict of interest.
